# Loop-mediated isothermal amplification identifies nematode *Leidynema* in the hindgut of non-pest cockroach

**DOI:** 10.1186/s13104-023-06467-z

**Published:** 2023-09-21

**Authors:** Laura J Schnell, Faraz Khan, Mel Hart, Maria C Davis

**Affiliations:** 1 Department of Biology, University of Regina, Regina, SK S4S 0A2 Canada; 2 Institute for Microbial Systems and Society, University of Regina, Regina, SK S4S 0A2 Canada; 3 Newark, DE 19713 USA

**Keywords:** *Blaberus*, *Leidynema appendiculata*, Parasitic nematode, Diet, LAMP, D2/D3 LSU sequencing

## Abstract

Cockroach microbiome studies generally focus on pest cockroach species belonging to the Blattidae and Ectobiidae families. There are no reports characterizing the gut microbiome of non-pest cockroach species *Blaberus discoidalis* (family Blaberidae), which is commonly used as a food source for insectivorous animals. We discovered the parasitic nematode *Leidynema appendiculata* in the *B. discoidalis* hindgut during initial work characterizing the gut microbiome of this organism. To determine the proportion of the *B. discoidalis* colony that was colonized by *L. appendiculata*, 28 S rDNA was amplified using two Methods: endpoint polymerase chain reaction (PCR) and loop-mediated isothermal amplification (LAMP). *B. discoidalis* colonies were raised on three diet types (control, high fibre, and high fat and salt) for 21 days before dissection. Each individual was sexed during dissection to identify potential sex-based effects of colonization. Data collected were analysed to determine if diet and sex impacted parasite colonization patterns. LAMP detected a higher proportion of parasite positive samples when compared to endpoint PCR. No sex- or diet-based differences in *L. appendiculata* colonization were found. This study adds to the limited existing knowledge of the *B. discoidalis* gut microbiome.

## Introduction

*Blaberus discoidalis*, commonly called the discoid cockroach or false death’s head cockroach, is a Central American cockroach belonging to the family Blaberidae. Discoid cockroaches are commonly used as nutrient rich food for insectivorous pets. In addition to being easily digestible due to low chitin content, these cockroaches are easily reared in homes since they are relatively odourless with limited motility when compared to other feeder insect species. However, little is known about the microfauna of discoid cockroaches, since most studies on cockroach gut microfauna focus on those of pest species such as *Periplaneta americana* (American cockroach) and *Blattella germanica* (German cockroach) [1–5]. Therefore, the composition of the parasitic (and/or commensal) interactions in the gut of discoid cockroaches, if any, remains unknown.

Parasitic nematode species from the order Oxyurida, commonly called “pinworms”, have been documented to form associations with several cockroach species [6]. Studies on the parasite prevalence in closely related pest cockroach species have concluded that thelastomatoid nematodes (super-family Thelastomatoidea) are endemic to these cockroaches [3, 5]. Among the thelastomatoid nematodes that have been categorized, only 22 species are endemic to cockroaches (order Blattodea) [7]. However, thelastomatoid species reported to date infect multiple hosts across orders, suggesting that host range in thelastomatoid nematodes is dependent on host ecology rather than host specificity [8]. Exploratory dissections of laboratory-reared discoid cockroaches prior to this study revealed the presence of adult nematodes in the digestive tract.

The aim of this study was to determine the prevalence of thelastomatoid nematodes in the discoid cockroach hindgut, and to determine if the nematode presence was related to arthropod sex or diet. Two methods were used to determine the presence of nematodes in the hindgut of *Blaberus discoidalis*: (1) endpoint PCR followed by 28 S rDNA Sanger sequencing, a technique often used to confirm the presence of nematodes belonging to the family Thelastomatidae [1, 9], and (2) loop-mediated isothermal amplification (LAMP). LAMP is both highly specific due to the use of four to six parasite-specific primers, and sensitive to low DNA concentration [10–12]. This allows for parasite DNA amplification with reduced bias for host 28 S rDNA. LAMP primers specific to the *L. appendiculata* 28 S rRNA gene were designed for use in the assay, while D2a and D3b primers [1] were used in the endpoint PCR assay to target the D2-D3 expansion of the 28 S rRNA gene. As expected, the LAMP assay detected more positive samples than endpoint PCR. Surprisingly, no sex- or diet-based changes in the prevalence of parasitic nematodes were found. This study provides a basis for the use of LAMP in the detection of cockroach nematodes and adds to sparse knowledge of the *B. discoidalis* hindgut microbiome.

## Methods

### Cockroach rearing and diet treatments

**Fig. 1:**
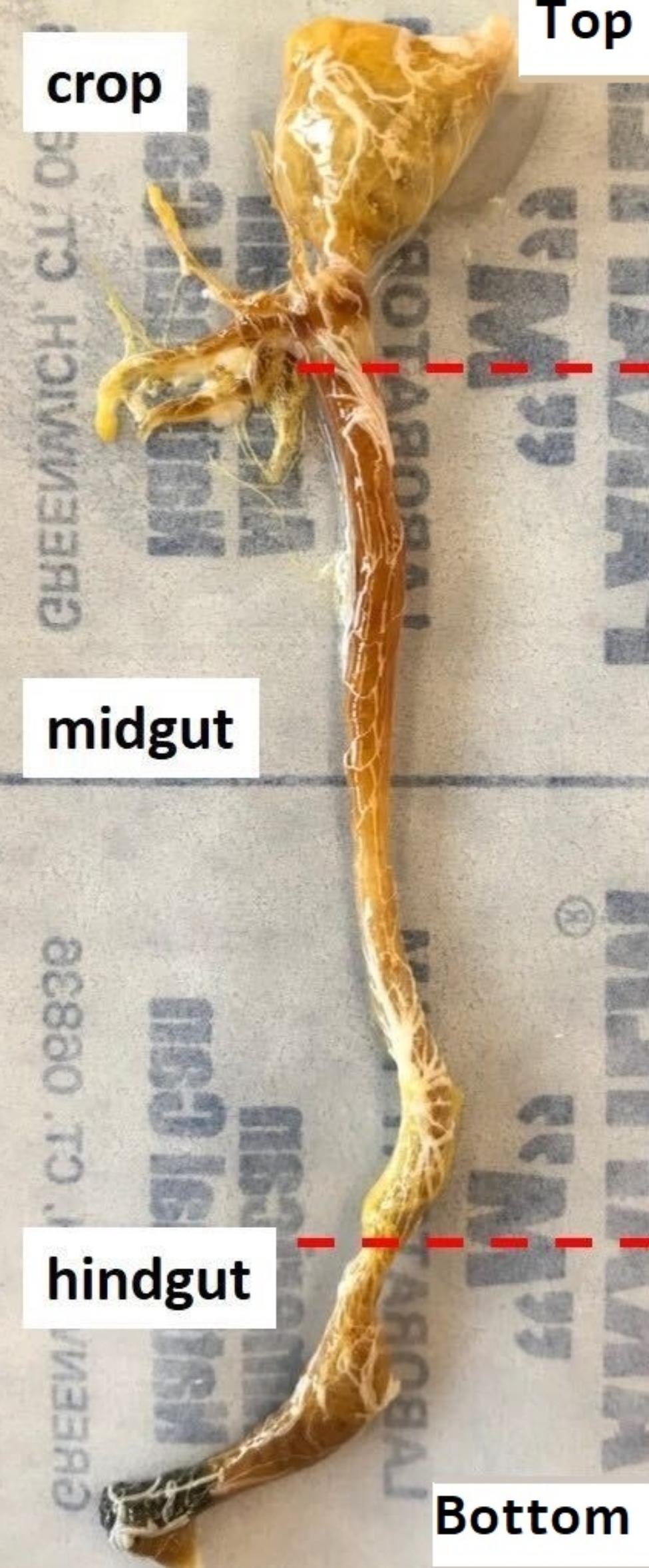
Image of a dissected *B. discoidalis* gut. The 3 sections (crop /foregut; midgut and hindgut) are sections are indicated red dashed lines

*Blaberus discoidalis* cockroaches were taken from a colony established in 2015 and kept at the University of Regina. Cockroaches were fed three different diet types: control (Vegetables and Pedigree dry dog food, Mattoon, Illinois, USA), high salt and fat (Old Dutch Cheese Pleesers; Winnipeg, Manitoba, Canada), and high fibre (sweet potato). Cockroaches were allowed to eat and drink water *ad libitum.* Cockroaches were kept in enclosures made of plexiglass, placed in natural light (May – June) at 21 °C and approximately 50% relative humidity. After 21 days of diet treatment, cockroaches were dissected. Cockroaches were anesthetized with ice and surface sterilized with 70% ethanol. Cockroaches were pinned with T pins and immobilized with leg cutting. A lengthwise cut was made on the ventral plane of the cockroach. The gut was removed after cuts were made in the esophagus and below the hindgut. The gut was divided into three sections: crop, mid- and hindgut (Fig. 1), separately flash-frozen in liquid nitrogen and stored at -80 °C until further processing.

DNA Extraction: DNA extraction protocol was adapted from Sambrook and Russel [13]. Thawed hindgut sections were treated with 500 µl of lysis buffer and incubated at 80 °C for 5 min. In the same tube, samples were homogenized. Proteins were precipitated with 200 µl of 7.5 mM ammonium acetate. DNA was precipitated using 100% (w/v) isopropanol and hydrated with 50 µl of 10 mM tris-HCl and stored at − 20 °C.

### Endpoint PCR (presence/absence) assay

Nematode presence was identified by amplifying the 28 S rRNA gene using universal primers D2a and D3b (Table 1) [1]. Products from endpoint PCR were gel-excised and purified using the QIAquick Gel Extraction Kit (QIAGEN Sciences, Maryland, USA) and sequenced (Centre d’expertise et de Services Genome Quebec, Canada). Sequences were identified using the National Centre for Biotechnology Information - Basic Local Alignment Tool (NCBI-BLAST; https://blast.ncbi.nlm.nih.gov/Blast.cgi). Approximately 20% of endpoint PCR samples were done in duplicate or triplicate (n = 25).

**Table 1:** Endpoint PCR and LAMP primers used to target parasitic nematode Leidynema appendiculata based on the 28 S rRNA gene. *Primers from [1]

Primer	Assay	Sequence (5’ -> 3’)
D2a*	28 S rDNA PCR	ACAAGTACCGTGAGGGAAAGTTG
D3b*	28 S rDNA PCR	TCGGAAGGAACCAGCTACTA
F3	28 S rDNA LAMP	CTGTCGTTGCTGGTTGCC
B3	28 S rDNA LAMP	AGCCGTTACGCTTACTAGCT
FIP (F1c-F2)	28 S rDNA LAMP	CACAGCGAATTTCTCAGCGCACTGGGTAGATGTTTGGTGGC
BIP (B1c-B2)	28 S rDNA LAMP	TCGCCTGATGCTTATGTGTCGGCTGCGAAGCAGCATCCAC
LF	28 S rDNA LAMP	GCAAATACACCAAGCACTGTCATC
LB	28 S rDNA LAMP	TGGTGAAGGCGTATCCGC

### Loop-mediated isothermal amplification (LAMP)

LAMP primer design was performed using the NEB LAMP Primer Design Tool (https://lamp.neb.com/#!/; New England Biolabs, Ontario, Canada). Primers (Table 1; Integrated DNA Technologies, Coralville, Iowa, USA) were designed to be specific to *L. appendiculata* and confirmed through NCBI Primer-BLAST (https://www.ncbi.nlm.nih.gov/tools/primer-blast/). *Leidynema appendiculata* 28 S rDNA sequences found in the *B. discoidalis* colony had single nucleotide polymorphisms (SNPs) in common with *L. appendiculata* strain KX (NCBI accession #KY057027) and closely related strains [14]. Segments with known SNPs were avoided during primer design to variable regions in the *L. appendiculata* 28 S rRNA gene. LAMP assays were performed using WarmStart® Colorimetric LAMP 2x Master Mix (New England Biolabs, Ontario, Canada) as per manufacturer instructions. A subset of samples (n = 24), including all initially negative samples (n = 13), were done in triplicate. Endpoint PCR using the LAMP outer primers (F3 and B3; Table 1) was also performed to confirm primer specificity. Select endpoint PCR products were purified (QIAquick PCR Purification Kit) and sequenced to confirm parasite identification. Additionally, outer primer PCR was done in triplicate on 12 samples.

### Data analysis

Data from LAMP and endpoint PCR were analyzed using R version 4.2.0 [15] with the following packages: forcats [16], tidyverse [17], and cowplot [18]. Logistic regression was done for both the LAMP and endpoint PCR data. A two-proportions z-test was done to compare the proportion of positive samples.

## Results and discussion

### Leidynema appendiculata confirmed in the hindgut of B. discoidalis

This work confirmed that the adult nematodes observed during exploratory dissections in the hindgut of *B. discoidalis* were *L. appendiculata*. Colorimetric LAMP found *L. appendiculata* in the hindgut of 96% of the sampled population regardless of diet treatment. This is consistent with studies done in pest cockroach species, which found *L. appendiculata* colonization in the majority of the cockroaches sampled [1; 9; 18]. *Leidynema appendiculata* colonization spreads through coprophagy, which is a *B. discoidalis* feeding strategy [1; 18]. When all members of a colony are practicing coprophagy in a contained environment, *L. appendiculata* colonization appears widespread.

### Sex and diet did not impact colonization

Logistic regression showed that none of the measured variables (sex, diet treatment, cage, and if the cockroach was gravid) significantly influenced *L. appendiculata* colonization. The lack of sex-based differences to colonization is consistent with studies done on *L. appendiculata* abundance in other cockroach species [3; 5]. However, the lack of diet-based differences seen in *L. appendiculata* colonization patterns was contrary to expectations. Previous research has shown that diet has an impact on host gut microbiome [9] and that *L. appendiculata* consume digestion end-products and gut bacteria [19]. Despite this, *L. appendiculata* appears able to tolerate all the diet treatments performed in this study.

### LAMP is a quick and effective method for nematode detection

**Fig. 2:**
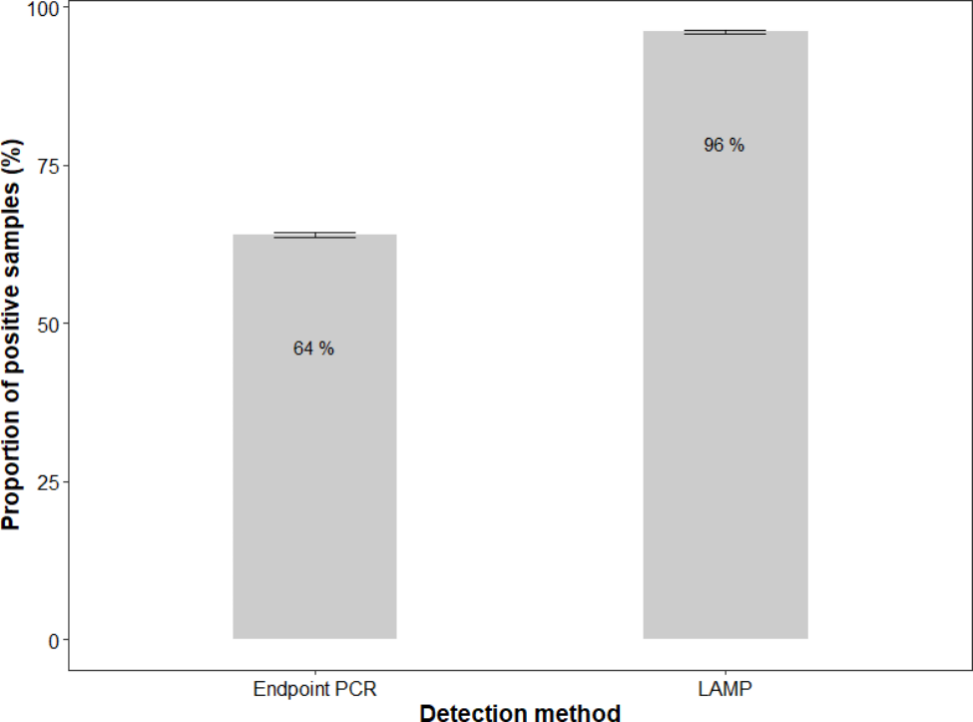
Comparison of the proportion of samples identified as positive for nematode presence by endpoint PCR and LAMP assays. Error bars represent standard error of the confidence interval obtained from a two-proportions z-test performed. The difference is statistically significant (p-value = 7.259e-10)

The samples tested using the LAMP assay had a 96% positive rate (n = 126) for nematode presence whereas only 64% of the samples tested with endpoint PCR using universal primers confirmed presence of *L. appendiculata* (n = 126) (Fig. 2). A two-tailed z-test showed this variance to be statistically significant (p-value = 7.259e-10). This difference is likely due to amplification bias of host rDNA, also amplified with the D2a/D3b universal primers. Host 28 S rRNA gene amplification was confirmed by Sanger sequencing and BLAST completed on endpoint PCR products. In addition, the LAMP assay is much less time consuming than endpoint PCR. Colorimetric LAMP produces results within 30–40 min. In contrast, PCR amplification (30 cycles) requires just over 1 h to amplify samples. This must be followed by visualization, which involves downstream analysis such as gel electrophoresis. This speed is one of the benefits of LAMP. However, this shorter amplification time can lead to false negatives in samples with low copy numbers.

### Limitations

LAMP has been shown to be highly effective for use in detecting certain pathogens such as SARS-CoV-2 [20]. However, some studies in parasite colonization have shown false negative results [21]. LAMP efficacy is highly dependent on primer design [21], which may be a contributing factor to studies where LAMP was found to be ineffective. Additionally, colorimetric LAMP methods may be less effective than real-time methods used in SARS-CoV-2 detection. In this study, LAMP was more effective than endpoint PCR using the universal D2a/D3b primers. However, we did observe cases of false negatives in the LAMP assay. A subset of the samples (n = 24), including all samples that tested negative (n = 13) using the LAMP assay, were done in triplicate to confirm the efficacy of LAMP as a detection method. All samples that tested positive for nematode presence in the initial LAMP assay also tested positive in subsequent runs. Of the 13 samples that tested negative for *Leidynema* presence in the initial LAMP run, eight samples tested positive in subsequent replications of the LAMP assay. All 13 samples initially identified as negative for nematode presence were also amplified with endpoint PCR using the *L. appendiculata*-specific LAMP outer primers in triplicate followed by Sanger sequencing to confirm nematode presence. Five samples consistently tested negative for nematode presence using the LAMP assay. Of these, three samples tested positive for *L. appendiculata* when using the nematode-specific outer primer endpoint PCR. These results demonstrate that false negatives can result when using the LAMP assay. We recommend performing replications on any samples that test negative using the LAMP assay and verifying using endpoint PCR with the parasite-specific LAMP outer primers to ensure the detection of false negatives. False positives are also a concern in this study. Nematode eggs are commonly found in the environment [22], and the presence of nematode eggs is not indicative of a parasitic infection. Because LAMP is a highly sensitive assay, some of the positives observed in this study may indicate DNA from eggs, not from an active infection, but from ingestion; cockroaches often participate in coprophagy as a feeding strategy [1; 18]. For this reason, any studies using LAMP for rapid identification of pathogens require the subsequent use of abundance determining techniques such as qPCR to quantify the abundance of the nematodes in the host gut.

## Conclusion

This study confirms the visual observation that the parasitic nematode *L. appendiculata* colonizes the hindgut of *B. discoidalis.* Contrary to previous findings for other cockroach species, this work found that there were no sex- or diet-based differences in nematode colonization of *B. discoidalis*. Although LAMP is a quick and efficient method for detection of specific DNA, this study shows that endpoint PCR using the nematode-specific outer primers designed for the LAMP assay can effectively be used to detect any false negatives that may result when using colorimetric LAMP assay.

## Data Availability

The datasets used and/or analysed during the current study are available from the corresponding author on reasonable request.
